# Disclosure of HIV seropositive status to sexual partners and its associated factors among patients attending antiretroviral treatment clinic follow up at Mekelle Hospital, Ethiopia: a cross sectional study

**DOI:** 10.1186/s13104-015-1056-5

**Published:** 2015-03-29

**Authors:** Minichil Genet, Girum Sebsibie, Teklemariam Gultie

**Affiliations:** Debre Tabor Health Science College, Debre Tabor, Amhara Regional State Ethiopia; College of Medicine and Health Science, Addis Ababa Science and Technology University, Addis Ababa, Ethiopia; College of Medicine and Health Science, Arba Minch University, Arba Minch, Ethiopia

**Keywords:** HIV status, Disclosure, Sexual partner, Mekelle, Antiretroviral treatment

## Abstract

**Background:**

Disclosure of human immune deficiency virus (HIV) positive status has a key role in the prevention and control of HIV/AIDS. The failure of people infected with HIV to disclose their positive status can expose their sexual partners to the virus. Identifying the factors associated with status disclosure is a priority issue as high proportion of people living with HIV do not discloses their status and to design appropriate strategy to deal with the issues this involves. The aim of this study was to assess the disclosure and its associated factors of HIV positive status to sexual partners among patients attending antiretroviral therapy (ART) clinic follow up at Mekelle Hospital, Tigray, Ethiopia.

**Methods:**

An institution based cross sectional study was conducted at Mekelle hospital. Samples of 324 individuals were selected by using systematic random sampling techniques from July 1st until the 30th July 2012. The data was collected by trained data collectors through pretested semi structured questionnaire. The collected data was cleaned, coded, entered and then analysed using SPSS version 16.0 windows program. Descriptive statistics, binary and multivariable regression analysis with 95% confidence interval was carried out and p value less than 0.05 used to determine the significant association.

**Results:**

A total of 324 people on ART care follow up were interviewed with 100% response rate. The overall HIV status disclosure to sexual partner was 57.4%. Among those who disclosed their HIV status 58.0% of them told their partner after one month of initial diagnosis. The study showed that there is significant association between knowing HIV status of sexual partner [AOR = 16.69, 95% CI (5.4, 51.65)], duration of HIV related care follow up [AOR = 5.48, 95% CI (2.17, 13.80)] and discussion before HIV testing [AOR = 4.33, 95% CI (1.43, 13.08)] with HIV positive status disclosure to sexual partner.

**Conclusions:**

A HIV positive status disclosure to a sexual partner in this study was lower than what was reported in other studies in Ethiopia. The duration of HIV related care follow up, knowing partners HIV status and prior discussion were the main factors which affect the practice of HIV positive status disclosure to their sexual partners.

## Background

HIV/AIDS pandemic is one of the most destructive epidemics in the history of humankind. Globally at the end of 2010, an estimated 34 million people were living with HIV, including 3.4 million children less than 15 years of age. In the same year, there were 2.7 million new HIV infections worldwide, including 390,000 children less than 15 years old. The annual number of people newly infected with HIV has risen in the Middle East and North Africa in 2010. The trends in AIDS related death also differ, in East Europe and Central Asia, the number of people dying from AIDS related cause increases more than tenfold between 2001 and 2010 [1].

One third (34%) of all people living with HIV in 2009 resided in the 10 countries in Sub- Saharan Africa and this included approximately 40% of all women living with HIV. It is estimated that 31% of the people newly infected with HIV and 34% of all the people dying from AIDS related causes in the same year lived in these 10 countries [2]. HIV disproportionately affects women and young people. Women accounts half (52%) of the global adult prevalence and 60% in sub-Saharan Africa [3].

In Ethiopia, the first case of HIV was reported in 1984. Since then, HIV/AIDS has become a major public health concern throughout the length and breadth of the country. According to the 2010 single point estimate, the estimated adult HIV/AIDS prevalence was 2.4% with an estimated 1.2 million people living with HIV/AIDS (PLWHA) in 2010 [4].

In Tigray region, a total of 75,120 peoples are living with HIV/AIDS and there are an estimated 3,686 annual AIDS deaths with the new HIV infection rate being 9,737, of these people 19,200 need ART [5]. In the region, the prevalence rate of HIV/AIDS was 1.8% in 2011, less than the national figure. According to the Ethiopian demographic and health survey (EDHS) 2011 report, the prevalence of HIV among couples was 1.7% and it is a sad reflection to note that most of these couples do not mutually know their HIV status [6].

The disclosure of a HIV status to a sexual partner can have varying effects. It may motivate partner for Voluntary Counselling and Testing (VCT), reduce risk behaviours, and increase acquisition of support and adherence to ART [7]. However, on the contrary, it may cause blame, discrimination, abandonment, depression and loss of economic support and disruption of family relationship. Therefore it is easy to see why some patients may not disclose their HIV positive status [8]. It can also then have a negative effect on women’s treatment out-comes which have improved so much in recent years. There are several factors associated with disclosure of HIV status such as not fearing negative outcomes of disclosure, having communication skills to disclose, having initiated antiretroviral therapy, and having ever seen People publicly disclose their HIV status [9]. In a study conducted in Tanzania a close relationship with the person they told, the need for help, and advice from VCT care providers were the factors which facilitate the disclosure of the HIV status [10]. In another study in Ethiopia several factors were mentioned as a reason for disclosure of HIV status such as educational status, and ART status [11].

So far, there is little known on HIV positive status disclosure in Tigray Region. This study assessed the magnitude and the factors affecting disclosure of HIV sero-positive status to sexual partners among patients attending ART clinic follow up at Mekelle hospital, Tigray, Ethiopia.

## Methods

### Study area, design and period

The study was conducted in Mekelle hospital using cross sectional study design from July 1st July 2012 until 30th July 2012.

### Sample size and sampling technique

The sample size was calculated by using single population proportion formula. The assumption includes prevalence of 69% of female HIV positive individuals disclosing their HIV positive status to their sexual partners in south west Ethiopia [12], 95% level of confidence and 5% marginal error and the final sample size was 324. There were a total of 3,500 patients registered for ART care in the hospital. Approximately 80 patients attend the PLWHA clinic each day. We invited every third patient attending the clinic to participate in an interview until the required sample of 324 was recruited. As patients attended the clinic on a monthly basis, no patients presented twice during the recruitment period. The study includes adult patients above the age of eighteen years old.

### Measurement

During the interview sexual partner was defined as any form of sexual relations including one night stand, casual partner, intimate lover, and spouse if married. Delayed disclosure was defined as disclosure of HIV status to sexual partner after one month of diagnosis.

### Data collection and analysis

A structured questionnaire was used on PLWHA attending the ART clinic and data was collected by two nurses working with in the clinic and supervised by the principal investigators. The questionnaire includes socio-demographic characteristics and the practice of HIV status disclosure of the study participants. The data collectors were trained for one day. The collected data was entered and analysed using SPSS version 16. Descriptive statistics and logistics regression was carried out to describe the variables and to determine their relationship with the outcome variable. Odds ratio with 95% CI at p < 0.05 was used to determine the significant level of association between predictors and outcome variable.

### Ethical considerations

The study was approved by the Ethical review committee of the college of Medicine and health science, Mekelle University. Oral consent was obtained from the study participants after detailed explanation about the objective of the study was explained in advance. Patients were interviewed in a strictly private room which was prepared near to ART clinic and all the information collected from the respondents was kept confidential.

## Results

### Socio-demographic characteristics of the study participants

A total of 324 people on HIV/AIDS care follow up were interviewed with 100% response rate. Among the participants 92.3% (299) and 92% (298) were urban resident and belong to Tigray ethnic group respectively. Female participants constitute 60.2% (195) and 45.1% (146) were in the age group of 35–44 years of the (Table 1).

**Table 1:** **Socio-demographic characteristics of the respondents**

**Variables**	**% (number)**
Residence	
Urban	92.3(299)
Rural	7.7(25)
Ethnicity	
Tigray	92.0(298)
Amhara	8.0(25)
Sex	
Female	60.2(195)
Male	39.8(129)
Age	
18-24	4.3(14)
25-34	38.9(126)
35-44	45.1(146)
45-54	9.6(31)
> = 55	2.2(7)
Occupation	
Daily worker	34.6(112)
Governmental employee	23.8(77)
House wife	18.2(59)
Merchant	17.0(55)
Farmer	3.4(11)
Student	3.1(10)
Marital status	
Married	49.4(160)
Separated	20.7(67)
Widowed	13.0(42)
Single	11.7(38)
Divorced	5.2(17)
Religion	
Orthodox	87.7(284)
Muslim	10.2(33)
Protestant	1.5(5)
Catholic	0.6(2)
Educational status	
Illiterate	24.0(78)
Primary school (1–4)	9.0(29)
Primary school (5–8)	30.6(99)
Secondary school (9–12)	23.1(75)
Twelve and above	13.3(43)
Monthly income	
<=500 Ethiopian birr	55.2(179)
>500 Ethiopian birr	44.8(145)

### HIV positive status disclosure practice

The disclosure of their HIV positive status was 57.4% (186) and out of these 58% (108) of them delayed their status disclosure until after one month of the initial diagnosis. Twenty nine percent of the respondents disclosed their status within one to two months and 13.4% after 6 months (Table 2).

**Table 2:** **HIV status disclosure and timing of the respondents among antiretroviral treatment clinic attendant at Mekelle Hospital, Tigray, Ethiopia**

**Disclosure status**	**Yes% (number)**	**No% (number)**
Disclosure to none	23.8(77)	76.2(247)
Disclosure to sexual partner	57.4(186)	42.6(138)
Disclosure to children	15.7(51)	84.3(273)
Disclosure to parents	8.6(28)	91.4(296)
Disclose within one month	29%(50)	
Disclose between 1–6 month	15.6%(27)	
Disclose after 6 months	13.4%(23)	

### Factors associated with disclosure of HIV positive status to sexual partner

The binary logistic regression analysis showed that living together with sexual partner [COR = 9.01, 95% CI (5.40, 15.02)] and knowing HIV status of sexual partners [COR = 38.21, 95% CI (19.67, 74.21)] were more likely to disclose their HIV seropositive status to their sexual partners. Educated participants [COR =1.95, 95% CI (1.17, 3.27)] and married respondents [COR = 8.95, 95% CI (5.34, 15.01)] were more likely to disclose their HIV status to sexual partner than their counterpart.

This study indicated that being on ART treatment [COR = 3.32, 95% CI (1.89, 5.83)], and having pre-test counselling [COR = 2.79, 95% CI (1.74, 4.47)] were more likely to disclose than their counterparts. There was a clear association between social related factors with disclosure to sexual partner. Being a member of HIV/AIDS association [COR = 2.09, 95% CI (1.29, 3.37)], disclosure HIV serostatus to peer groups and relatives [COR = 2.16, 95% CI (1.36, 3.43)], seeing a person who discloses HIV status to the community [COR = 2.38, 95% CI (1.52, 3.75)], and those in a stable relationship with their sexual partner before HIV test were significantly associated with disclosure to sexual partner.

After controlling multiple confounding factors by using multiple logistic regressions, variables which showed independent effect on disclosure of HIV status to sexual partner were identified. The result showed that patients knowing their sexual partners HIV status were more likely to disclose than those who did not knew their partner’s HIV status [AOR = 16.69, 95% CI (5.4, 51.65)]. Those who had prior discussions before testing with their sexual partner were disclose their HIV status to sexual partner than those who did not discussed the situation [AOR = 4.3, 95% CI (1.43, 13.08)]. From different health care related factors only those who have had HIV related care follow-up more than two years were more likely to disclose to their sexual partner than those who had less than two years follow up [AOR = 5.48, 95% CI (2.17, 13.80)] (Table 3).

**Table 3:** **Predictors of HIV status disclosure to sexual partners among antiretroviral treatment clinic attendant at Mekelle Hospital, Tigray, Ethiopia**

**Variables**	**Disclosure status to sexual partner**		**COR (95% CI)**	**AOR (95% CI)**
	**Yes**	**No**		
Marital status				
Married	131 (81.9%)	29 (18.1%)	8.95 (5.34, 15.00)	
Unmarried	55 (33.5%)	109 (66.5%)	1	
Educational status				
Illiterate	35 (44.9%)	43 (55.1%)	1	
Literate	151 (61.4%)	95 (38.6%)	1.95 (1.17, 3.27)	
Residing with sexual partner				
Yes	136 (81%)	32 (19%)	9.01 (5.40, 15.02)	
No	50 (32.1%)	106 (67.9%)	1	
Knowing partners HIV status				
Yes	151 (91.5%)	14 (8.5%)	38.21 (19.68, 74.21)	16.69(5.4, 51.65)*
0.3 (0.1,1.3)		124 (78%)		
No	35 (22%)		1	1
Duration of HIV related care				
≤2 years	65 (43%)	86 (57%)	1	1
>2 years	121 (69.9%)	52 (30.1%)	3.08 (1.95, 4.87)	5.48 (2.17, 13.80)*
Prior discussion before test				
Yes	122 (91.7%)	11 (8.38%)	22.0 (11.08, 43.72)	4.33(1.43, 13.08)*
No	64 (33.5%)	127 (66.5%)	1	1
Relation before test				
Smooth	171 (67.3%)	83 (32.7%)	4.81 (2.49, 9.30)	
Disagreement	15 (30%)	35 (70%)	1	
Pre-test counseling				
Yes	140 (66%)	72 (34%)	2.80(1.74, 4.50)	
No	46 (41.1%)	66 (58.9%)	1	
Member of HIV/AIDS club				
Yes	79 (68.7%)	36 (31.3%)	2.09 (1.30, 3.38)	
No	107 (51.2%)	102 (48.8%)	1	

*Significant at p-value <0.05.

## Discussion

A disclosure of seropositive status to sexual partners enables the couples to make informed reproductive health choices that may ultimately lower the number of unintended pregnancies among HIV positive individuals, and reduce the risk of HIV transmission from mother to child. Similar to other studies, most of this study subjects were in the young age group which is due to the fact that HIV affects young age people.

The level of disclosure to a sexual partner was low (57.4%) and about 33.4% of the participants disclosed their HIV status after one month of diagnosis. It is lower than the finding in Jimma university hospital (90.8%), Hawasa referral hospital (85.7%), Kemissie Northeast Ethiopia (93.1%) and North West Ethiopia (69%) [12-16]. The reasons for these variations might be due to the difference in the study population socio-demographic characters; as this study was conducted in clinical setting and include all PLWHA and having sexual partner even at one time whereas some of other studies included only currently couple individuals.

In this study, it was observed that prior discussion, knowing partner status, and duration of HIV related care follow up were significantly associated with disclosure of HIV positive status to sexual partner after adjusting for confounding variables. This finding is similar with other studies [12,13,15,17-19]. Communicating with one’s partner prior to HIV testing is a key point in that it might help individuals to anticipate a partner’s reaction and would give them an opportunity to raise the issue and disclose their result. In this particular case disclosure may be easily discussed between partners as the conversation had been commenced before testing.

The duration of HIV care follow up of more than two years was associated with disclosure of HIV status to sexual partner and this finding is similar with other studies done in Jimma and Southern Ethiopia [13,14]. This similarity could be the result of a continuous counselling at each contact of health professionals and use of behaviour rehearsal technique which is aimed at helping patients to develop healthy behaviours including disclosure of HIV positive status to sexual partner.

This study indicated that there is a relationship between ART initiation and disclosure of HIV status to sexual partner. This finding is similar to a qualitative study in Tanzania on PLWHA and also a study done in Mozambique showed that 59.5% males and 48.7% females disclose their HIV test result to sexual partner after 12 month of initiation of HAART [20-22]. This discrepancy might be PLWHA on ART often reported feeling comfortable with their status. This was seen as a result of overcoming internalized feelings of shame facilitated disclosure of HIV status. The other possible reason may be people on ART receive pre ART counseling where disclosure is emphasized as a precondition and requirement for starting the treatment.

Consistent to other findings, in this study, married women were more likely to disclose HIV positive status to sexual partners [23]. This could be due to intimacy of partners; strength of their relationship, feeling of responsibility and the confidence they have with each other, which would facilitate open communication which in turn caused to disclose their status. On the other hand individuals residing together with their sexual partner were also associated with disclosure of HIV status like other similar finding observed in Ethiopia and Rwanda [14,24].

The finding of this study showed that there is an association between having normal healthy relationship and HIV status disclosure to sexual partner but not statistically significant like other studies [15]. This association might be due to the reason that in this study all types of sexual relations were included unlike to the other studies which only focus on long term committed partners.

Discussion about HIV testing and test result with friends and relatives also facilitated disclosure of HIV positive result to sexual partner, this finding also similar to that of Kemissie studies on PLWHA adult clinical service users [15]. This might have helped individuals to be strong and disclose their result to their partner. This may be because sharing ideas with friends and relative gave strength for individuals spiritually as well as mentally, so that they can anticipate and accept the outcomes following disclosure.

However, this study has some limitations such as disclosure status was assessed through self-reporting questions, and this study utilized cross sectional study design which made it impossible to establish a causal relationship between the outcome and exposure variables.

## Conclusions

The HIV positive status disclosure to sexual partner in this study is low. Two third of the study participants disclosed their HIV status to their sexual partners after a month, yet the majority of the respondents were sexually active. Generally disclosure of HIV status to a sexual partner was strongly associated with; prior discussion about HIV before testing, knowing the status of their sexual partners and duration of HIV related care follow up for more than two years.

## Abbreviations

AIDS: Acquired Immune Deficiency Syndrome
HIV: Human Immune Virus
PLWHA: People living with HIV/AIDS
HAART: Highly Active Antiretroviral Treatment
